# National mapping of soil-transmitted helminth and schistosome infections in Ethiopia

**DOI:** 10.1186/s13071-020-04317-6

**Published:** 2020-09-01

**Authors:** Gemechu Tadesse Leta, Kalkidan Mekete, Yonas Wuletaw, Abeba Gebretsadik, Heven Sime, Sindew Mekasha, Adugna Woyessa, Oumer Shafi, Jozef Vercruysse, Jack E. T. Grimes, Iain Gardiner, Michael French, Bruno Levecke, Lesley Drake, Wendy Harrison, Alan Fenwick

**Affiliations:** 1 Ethiopian Public Health Institute, PO Box 1242/5654, Addis Ababa, Ethiopia; 2 Department of Virology, Parasitology and Immunology, Faculty of Veterinary Medicine, Ghent University, Merelbeke, Belgium; 3 Federal Ministry of Health, PO Box 1234, Addis Ababa, Ethiopia; 4 Department of Civil and Environmental Engineering, South Kensington Campus, Imperial College London, London, SW7 2AZ UK; 5 Partnership for Child Development, Department of Infectious Disease Epidemiology, St Mary’s Campus, Imperial College London, London, W2 1PG UK; 6 Schistosomiasis Control Initiative, Department of Infectious Disease Epidemiology, St Mary’s Campus, Imperial College London, London, W2 1PG UK; 7 RTI International, Washington D.C, USA

**Keywords:** Soil-transmitted helminthiasis, Schistosomiasis, *Ascaris*, *Trichuris*, hookworms, *Schistosoma mansoni*, *Schistosoma haematobium*, Woreda (districts), Disease maps, School-aged children, Endemicity, Mass drug administration

## Abstract

**Background:**

An accurate understanding of the geographical distributions of both soil-transmitted helminths (STHs; *Ascaris lumbricoides*, *Trichuris trichiura*, and the hookworms *Necator americanus* and *Ancylostoma duodenale*) and schistosomes (SCH; *Schistosoma mansoni* and *S. haematobium*) is pivotal to be able to effectively design and implement mass drug administration (MDA) programmes. The objective of this study was to provide up-to-date data on the distribution of both STH and SCH in Ethiopia to inform the design of the national control program and to be able to efficiently achieve the 75% MDA coverage target set by the WHO.

**Methods:**

Between 2013 and 2015, we assessed the distributions of STH and SCH infections in a nationwide survey covering 153,238 school-aged children (aged 5–15 years), from 625 woredas (districts), representing all nine Regional States and two City Administrations of Ethiopia. Nationwide disease maps were developed at the woreda level to enable recommendations on the design of the national MDA programme.

**Results:**

The prevalence of any STH infection across the study population was 21.7%, with *A. lumbricoides* (12.8%) being the most prevalent STH, followed by hookworms (7.6%) and *T. trichiura* (5.9%). The prevalence for any SCH was 4.0% in areas where both SCH species were evaluated. *Schistosoma mansoni* was the most prevalent SCH (3.5 *vs* 0.3%). STHs were more prevalent in southwest Ethiopia, whereas SCH was found mostly in the west and northeast of the country. The prevalence of moderate-to-heavy intensity infections was 2.0% for STHs and 1.6% for SCH. For STH, a total of 251 woredas were classified as moderately (*n* = 178) or highly endemic (*n* = 73), and therefore qualify for an annual and biannual MDA program, respectively. For SCH, 67 woredas were classified as endemic and 8 as highly endemic, and hence they require every two years and annual MDA programme, respectively.

**Conclusions:**

The results confirm that Ethiopia is endemic for both STHs and SCH, posing a significant public health problem. Following the WHO recommendations on mass drug administration, 18 and 14 million school-aged children are in need of MDA for STHs and SCH, respectively, based on the number of SACs that live on the eligible geographical areas.
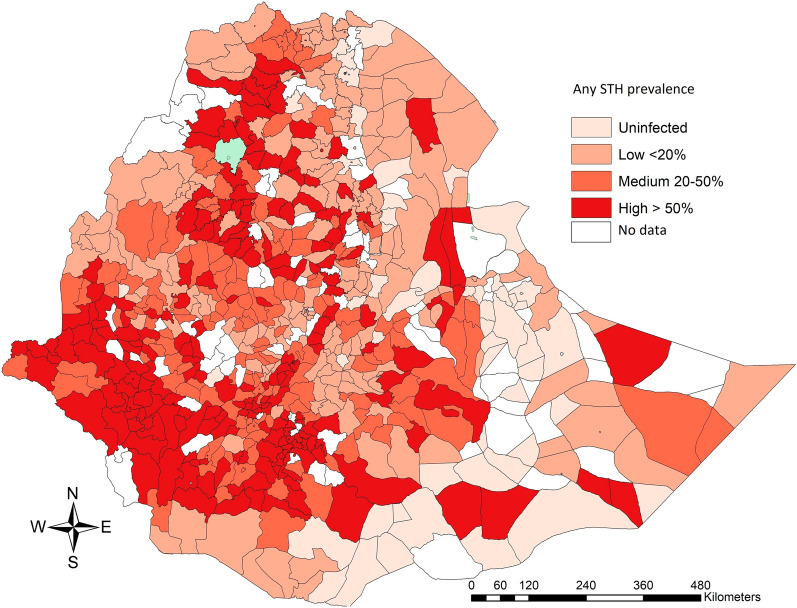

## Background

Neglected tropical diseases (NTDs) are a diverse group of bacterial, parasitic and viral communicable diseases. They are widespread in tropical and subtropical countries where poverty, inadequate sanitation and hygiene are common [1]. A group of these NTDs can be controlled by either innovation or intensified disease management at the level of the individual patient, or through mass drug administration (MDA) to the most at-risk populations. Some of the MDA-amenable parasites include the soil-transmitted helminths (STHs): *Ascaris lumbricoides*, *Trichuris trichiura* and the hookworms (*Nectator americanus* and *Ancylostoma duodenale*) and schistosomes (SCH: *Schistsoma* *mansoni* and *S. haematobium*). The MDA platforms for these NTDs are often based on deworming children at school. The recommended dosages for deworming are a single oral dose of albendazole (400 mg) or mebendazole (500 mg) for STHs, and praziquantel for SCH (40 mg/kg; the weight often approximated by height using a dose-pole [2]). The global community has committed to cover at least 75% of the school-aged children (SAC) in endemic areas for both STHs and SCH by 2020 [3], with the ultimate goal to reduce the prevalence of heavy intensity (HI, in case of SCH) or moderate-to-heavy intensity (MHI, for STH) infections, as defined by the WHO [4]. These are thought to be the intensity of infections that cause the highest morbidity. The frequency and populations targeted for MDA are determined by the prevalence in each implementation unit. For STHs, it is recommended to distribute drugs twice a year when the prevalence of any STHs exceeds 50%, and once a year when the prevalence is at least 20%. For prevalence below 20%, a case-by-case treatment is recommended [4]. For SCH, an annual round of MDA is recommended when the prevalence is ≥ 50%, every two years when the prevalence is ≥ 10%. For any other prevalence of ≥ 1%, twice MDA during the primary school years is recommended [2].

Both STHs and SCH infections pose an important threat to public health in Ethiopia [5–9]. In 2012, it was concluded that Ethiopia represents one of the top five sub-Saharan countries with the highest prevalence of STHs (second place for *Ascaris*, third place for hookworm and fourth for *Trichuris*). For SCH, Ethiopia has the 14th highest prevalence among sub-Saharan countries [10]. Across the country, there have been a series of disease distribution studies, reporting a wide range of infection [5, 6, 9, 11, 12]. However, these studies are not ideal for making recommendations on the best MDA strategy at a national level. They are generally dated (early 1970s to 2015; [5, 9, 13]), nor do they cover the entire country. Moreover, they differ considerably in sample size, design and diagnostic method utilized hindering direct comparison [9, 12, 14, 15], which further complicates effectively designing and implementing MDA programs. Since 2000, the Ethiopian government has implemented STH deworming of children in under five as part of an integrated vitamin A supplementation programme. For SCH, the country has historically focused on a case-based treatment of laboratory-confirmed patients. Following the London Declaration on NTDs in 2012 [16], the Federal Ministry of Health of Ethiopia (FMoH) developed an NTD Master Plan and Roadmap for combating the country’s most common NTDs [17]. For STHs and SCH, the target is to control morbidity through the reduction of MHI infections by means of MDA. Up-to-date data on disease distribution are therefore pivotal to ensure the area’s most in need of MDA are covered and to avoid the initiation of large-scale deworming in areas where disease is rare or absent [4, 18].

Here, we describe the distribution of STHs and SCH in 153,238 SAC, across 2989 schools in 625 woredas, across all nine Regions and two City Administrations in Ethiopia. We present disease distribution maps to inform the design of the national programme.

## Methods

### Study area

In 2015, the Ethiopia’s population was estimated at 100 million [19]. The country has three administrative levels. The first level includes nine Regional States: Afar, Amhara, Benishangul-Gumuz, Gambela, Harari, Oromia, Southern Nations, Nationalities and Peoples’ Region (SNNPR), Somali and Tigray; and two chartered City Administrations: Addis Ababa and Dire Dawa. The second and the third administrative levels are zones and woredas (districts), respectively. Woredas are the implementation units for NTD control programmes in Ethiopia.

The distribution of STHs and SCH was mapped over two surveys. In the first survey (November 2013 to March 2014), eight of the nine Regional States and one of the two City Administrations were included. In the second survey (February and April 2015), Amhara and Addis Ababa were mapped. In addition, the second survey included fine-scale mapping survey in Somali Regional State with the aim to cover woredas that were not mapped in the first survey.

### Field procedures

For each woreda (or sub-city in the case of City Administrations), ten schools were randomly selected from the list of elementary schools provided by the Federal Ministry of Education (FMoE). From these, five schools were purposively selected by the Woreda Health Office, biased towards schools thought to be at-risk for SCH infections (due to proximity to water bodies, reports on SCH infections, irrigation and fishing practices of the community). An exception to this rule were those woredas where the safety of the field teams during the survey could not be secured. This was of particular impact in Somali Region and was compounded by the relatively small numbers of children enrolled per school. This is evidenced in the number of people surveyed. For the later determination of treatment approach, in these areas the nearest adjacent woreda was used to decide MDA.

Once the school selection was completed, the field team made the necessary pre-visit arrangements with the school directors. On the day of visit, all students of grade 5 (children around 12 years of age) were arranged in two lines, one for girls and one for boys. A random selection was made of 25 girls and 25 boys, resulting in a total maximum of 50 students per school. In schools with fewer than 25 boys or girls in the appropriate grade, children from lower grades (grade four: children around 11 years of age) or higher grade (grade six: children around 13 years of age) were included. The selected students were asked to provide both a stool and a urine sample.

Stool samples were screened for the presence of STHs and *S. mansoni* eggs, applying a single Kato-Katz thick smear [20]. The number of eggs of STHs (*A. lumbricoides*, *T. trichiura* and hookworms) and *S. mansoni* were multiplied by 24 to obtain the faecal egg counts (FECs) expressed in eggs per gram of stool (EPG) for each of the four helminth species.

Urine specimens were screened for *S. haematobium* in two consecutive steps. First, the presence of haematuria was assessed using Haemastix® (Bayer HealthCare LLC, Elkhart, Indiana, USA). The results of this test were recorded as either negative, trace, +, ++ or +++. Subsequently, urine samples in which at least a trace of haematuria was detected, were subjected to urine filtration to assess the number of *S. haematobium* eggs in 10 ml of urine.

To ensure the quality of the parasitological results, the field team were instructed to read slides within 30 min to avoid over-clearing of hookworm eggs. For each team, we assigned a team leader to read 10% of the Kato-Katz slides and give feedback on a timely basis to the field team. Given challenges of hookworm egg degradation with time, hookworm eggs were not used for quality control purposes.

In addition, a questionnaire was conducted to collect information on water, sanitation and hygiene (WASH) at the school level. The results of this questionnaire and parasite infection for the first round of survey were reported elsewhere by Grimes et al. [21].

### Training of the field teams

In total, 54 field teams from the different Regional States and City Administrations were involved in this survey. Each team consisted of one health officer (for the questionnaires and treatment) and three laboratory technicians (for the examination of stool and urine samples). The training was provided at the Ethiopian Management Institute (EMI) in Bishoftu, Oromia regional state. During these trainings, both theoretical and practical sessions were given to 164 health officers and laboratory technicians. The theoretical sessions focussed on a variety of aspects of the diseases (life-cycles, pathogenesis, laboratory diagnosis, prevention and control strategies), while the practical sessions focussed on the diagnostic methods (the use of Haemastix®, urine filtration and Kato-Katz thick smear), archiving Kato-Katz slides for quality control, completing questionnaires on WASH and using LINKS^®^ (a smart phone-based application to collect data).

### Mapping coordination and supervision

Additional file 1: Figure S1 provides an overview of the coordination and supervision of this study. The Ethiopian Public Health Institute (EPHI), the technical arm of FMoH, was responsible for the overall coordination of the survey. For this coordination, four central supervisors were assigned. At the Regional States and City Administrations, at least one supervisor from either the Regional Health or Education Office for each Regional State (and City Administration) was assigned throughout the mapping period. In addition, external supervisors from the Ugandan Vector Control Division (Uganda) and the Kenyan Medical Research Institute (Kenya) conducted monitoring visits. These supervisors independently evaluated the survey coordination and communication flow between the central level (EPHI), the regional supervisors (Regional States and City Administration) and the field teams. They also monitored the operational procedures at the schools, providing feedback to the field teams, correcting those that were not adhering to the mapping protocol.

### Data collection and data management

Data were collected using the LINKS® data collection system developed by the Task Force for Global Health (Atlanta, USA). This is an android-based application that allows standardized entry of epidemiological data across the different teams. At the school level, the teams collected the GPS coordinates, the total number of students, the total number of boys and girls in the school, the availability of toilets, water supplies and hand washing facilities. At the individual level, the teams collected the age, sex, the number of *Ascaris*, *Trichuris*, hookworm, *S. mansoni* eggs, the presence of haematuria, and the number of *S. haematobium* eggs. Raw data were downloaded from the LINKS system server and were subsequently curated using Microsoft Excel (Microsoft Corporation, Redmond, WA, USA).

### Statistical data analysis

The prevalence and intensity of infections were calculated for any STH and SCH, and the individual helminth species, separately. Prevalence was estimated by the proportion of children for whom eggs of a particular helminth species were detected. Intensity of infection was measured as the prevalence of MHI infections were calculated for any STH and SCH, and for the different helminth species separately [4]. Unweighted prevalence was calculated at both regional and woreda level. To this end, the proportion of children excreting eggs over the total number screened in the region or woreda was calculated. Subsequently, the point estimates of the different parameters were plotted on a geographical map of Ethiopia using ArcGIS version 10.4 (ESRI, Inc., Redlands, USA).

These infection parameters (prevalence, infection intensity and mixed infections) were calculated at the different administrative levels (national, Regional States/City Administrations, woredas and schools). Subsequently, the point estimates of the different parameters were plotted on a geographical map of Ethiopia using ArcGIS version 10.4 (ESRI, Inc.). We explored the variation in infections of any intensity and MHI by generalized linear mixed models, which were fitted for each of the five helminth species with the presence/absence of infections of any intensity or MHI infections as outcome, the age (in years), sex (2 levels: boy and girl) and Region/City Administration (11 levels: Afar, Amhara, Benishangul-Gumuz, Gambela, Harari, Oromiya, Southern Nations, Nationalities and Peoples’ Region (SNNPR), Somali, Tigray (Regions), Addis Ababa and Dire Dawa (City Administrations)) as explanatory variables. In the analysis, we accounted for clustering of children from the same school and schools from the same woreda. To facilitate an easy interpretation, we centralized the age around its median (12 years) and converted into a binary outcome (boy = 0, girl = 1). The level of significance was set at *P* < 0.05.

In addition, we determined the prevalence of mixed infections (the proportion of children who were excreting eggs of at least two different helminths). Finally, we determined the MDA design for each of the woredas included in this survey. To this end, we classified the woredas into low, moderate and high endemic for STHs and SCHs applying the WHO classification criteria [4] on the prevalence of any STH and SCH infection across the five schools of the same woreda (the proportion of cases over all children screened in that woreda).

### Quality control result analysis for the Kato-Katz thick smear

Quality control of the FECs of any STHs and *S. mansoni* was performed for 6042 individuals. For this, Kato-Katz thick smears were re-examined by a team leader. The proportions of both false positives and false negatives were assessed. To this end, we assumed that the team leader was correct. In cases where both results indicated presence of eggs, the agreement in egg counts was assessed. Result disagreements were defined when the difference in egg counts was greater than 10 when the team leader counted fewer than 100, or when the difference in egg counts was more than 20% when the team leader counted more than 100 eggs [22].

### Map creation

The results from the field surveys were used to develop graphical maps of the distribution of SCH and STH infection, and of SCH and STH moderate and high intensity infections. This was done in ArcGIS version 10.4 (ESRI, Inc.). The maps are intended as a useful geographical guide of the distribution of infection. There were challenges with representing the woreda-level results from the field surveys onto a graphical map, meaning a one-to-one representation was not possible. This was related particularly to the rapid increase in the number of woredas in the country (resulting in splits between ‘mother’ and ‘daughter’ districts and subsequent changes to administrative boundaries) and the lag in availability of updated mapping software to represent these accurately. This means that the graphical maps cannot be a perfect one-to-one representation of the data collected. This is an inherent challenge in countries with changing administrative borders and sub-divisions. The numbers of woredas in the tables for each infection category are most accurate and used to inform the control program.

### Informing MDA strategy

In order to determine the MDA strategy in each district, the upper 95% confidence interval of woreda (district) level prevalence was taken of either ‘any STH’ or ‘any SCH’. This was then compared to the WHO-recommended cut-offs [2]. Using the upper confidence interval rather than the point prevalence represents a conservative approach to ensure the program reaches as many people in need of treatment as possible.

## Results

### Study population

Stool samples were collected from 153,238 SAC from 2989 schools in 625 woredas across the nine Regional States and two City Administrations for both STH and *S. mansoni* infections. Urine samples were also collected from 112,218 SAC attending 2226 schools in 466 woredas across the 9 Regional States and 2 City Administrations. In both datasets, the age of the children ranged from five to 15 years, with a median age of 12 years. The sexes were generally equally represented (ratio of males/females = 0.99).

In the following paragraphs we summarize the prevalence of infections of any intensity and MHI for STHs and SCH, separately. Then we discuss the occurrence of mixed STH and SCH infections. Finally, we provide an overview of the recommended MDA strategy at the level of the woreda for both STHs and SCH.

### Prevalence of STH infections of any intensity and MHI

**Fig. 1:**
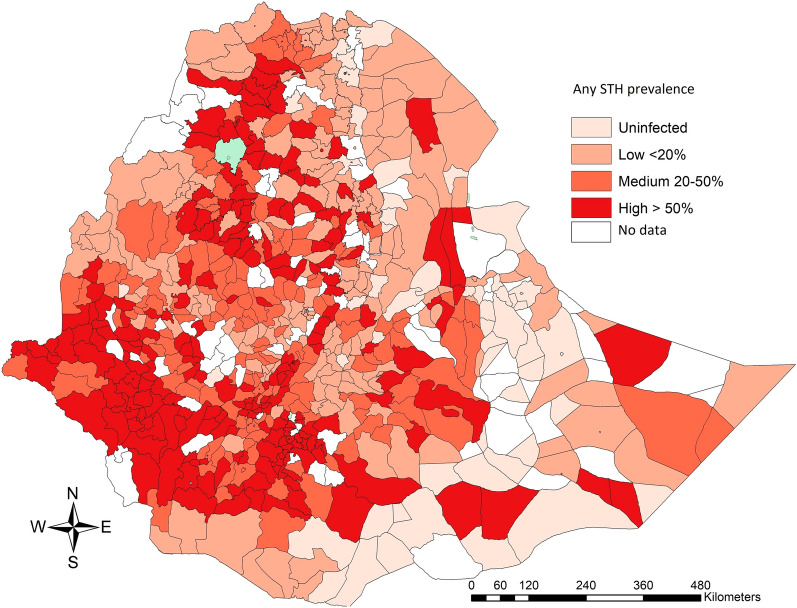
The woreda prevalence of any soil-transmitted helminth infections in school-aged children, Ethiopia 2013–2015. The map illustrates the woreda-level prevalence of any soil-transmitted helminth infections in 153,238 school-aged children, Ethiopia 2013–2015. The woreda-level prevalence is based on the number of children, which were found to be positive based on single Kato-Katz thick smear, over the total number of children screened in a woreda. The source of the administrative boundaries is www.gadm.org/

The unweighted prevalence of STH infections of any intensity and MHI intensity across the Regions/City Administrations, sex and age are summarized in Table 1. The overall unweighted prevalence of infection with any STH was 21.7%. The most prevalent STH species was *A. lumbricoides* (12.8%), followed by hookworms (7.6%) and *T. trichiura* (5.9%). There was a large variation in prevalence across the different Regional States and City Administrations. For any STH infection, the prevalence ranged from 1.7% (Addis Ababa City) to 58.1% (Gambela). For the different STH species separately the prevalence ranged from 0.1% (Harari) to 45.2% (Gambela) for *Ascaris*, from 0.0% (Addis Ababa City Administration) to 21.5% (Gambela) for hookworms, and from 0.1% (Afar, Dire Dawa City Administration and Harari) to 15.3% (SNNPR) for *T. trichiura.* Figure 1 illustrates the prevalence at the woreda level for any STH infection. In general, the distribution of any STH infection is more prevalent in the south (SNNPR), southwest (Gambela, Oromiya and Benishangul-Gumuz) and north (Amhara and Tigray) of Ethiopia and less prevalent in the east of the country (Afar).

**Table 1:** Variation in prevalence of soil-transmitted helminth infections across regions/city administrations, sex and age

	*n*	Any STH (%)		*A. lumbricoides* (%)		*T. trichiura* (%)		Hookworm (%)	
		Any intensity	MHI	Any intensity	MHI	Any infection	MHI	Any infection	MHI
Region/City Administration									
Addis Ababa	2718	1.7	0.0	1.2	0.0	0.6	0.0	0.0	0.0
Afar	2377	2.2	0.0	2.0	0.0	0.1	0.0	0.2	0.0
Amhara	25,246	14.3	0.5	8.0***	0.4	0.6	0.0	6.5***	0.0
Benishangul-Gumuz	3109	10.3	0.0	0.8	0.0	0.7	0.0	9.3***	0.0
Dire Dawa	1701	2.3	0.0	1.6	0.0	0.1	0.0	0.8	0.0
Gambela	2977	58.1	0.6	45.2***	0.6	6.3	0.0	21.5***	0.1
Harari	1804	3.0	0.0	0.1**	0.0	0.1	0.0	2.8*	0.0
Oromia	62,520	16.7	1.2	9.3***	0.9	4.6**	0.2	5.6***	0.2
SNNPR	37,544	42.4	5.7	25.9***	5.2	15.3***	0.3	13.5***	0.2
Somali	966	38.0	0.0	36.4***	0.0	1.2	0.0	5.5***	0.0
Tigray	12,276	5.4	0.1	2.2	0.0	0.2	0.0	3.3***	0.0
Sex									
Sex 0 (male)	76,385	21.6	1.9	12.8	1.7	5.9	0.1	7.6	0.1
Sex 1 (female)	76,853	21.8	2.0	12.8	1.8	5.9	0.2	7.6	0.1
Age (in years)									
5–9	1201	21.8	0.3	12.8	0.2	4.7	0.1	8.5	0.1
10	23,962	21.2	1.7	11.8	1.4	5.8	0.1	8.0	0.2
11	38,332	21.1	1.8	12.1	1.6	5.9	0.2	7.4	0.1
12	45,447	21.8	2.3	13.0	2.0	6.2	0.1	7.3	0.1
13	27,693	22.3	2.2	13.5	1.9	5.9	0.2	7.7	0.1
14	15,342	22.0	1.6	13.4	1.4	5.2	0.1	8.1	0.1
15	1261	30.3	2.6	22.7	2.4	7.9	0.2	8.3	0.1
Total	153,238	21.7	2.0	12.8	1.7	5.9	0.2	7.6	0.1

*Notes*: MHI: moderate-to-heavy intensity, comparisons to the baseline value (Region/City Administration: Afar; sex: sex 0; age = 12 years) that reveal an odds ratio significantly different from one are indicated by at least one asterisk

*P < 0.05, **P < 0.01, ***P < 0.001

The variation in any STH infections across sex was small. For example, the prevalence of any STH infections was equally distributed across both sexes (21.6% for sex = 0 *vs* 21.8% for sex = 1), and this absence of any variation across sex was also observed for the different STH species, separately (*Ascaris*: 12.8 *vs* 12.8%; *Trichuris*: 5.9 *vs* 5.9%; hookworms: 7.6 *vs* 7.6%). The variation in prevalence was more clear across the different age categories. The prevalence of any STH infection ranged from 21.1% in SAC of 11 years-old to 30.3% in SAC of 15 years-old. Across the different STH species, this distinct variation was only observed for *Ascaris* infections (SAC of 11 years-old: 11.8% *vs* SAC of 15 years-old: 22.7%). For the other STH species, the variation in prevalence was less pronounced, the difference in prevalence across the age categories not exceeding 3.5 percentage points (*Trichuris*: 4.7 *vs* 7.9%; hookworms: 7.3 *vs* 8.5%).

**Fig. 2:**
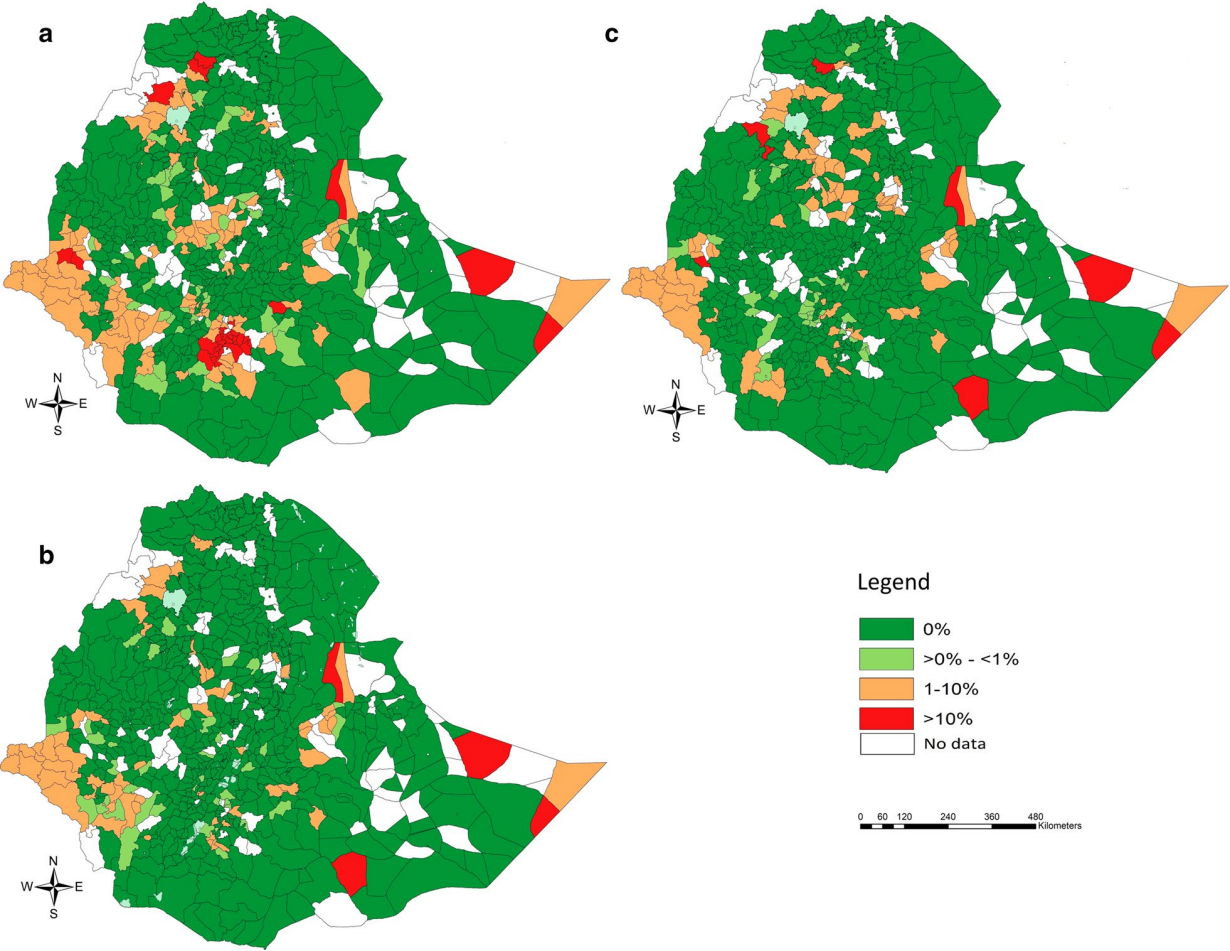
The woreda prevalence of moderate-to-heavy intensity soil-transmitted helminth infections in school-aged children, Ethiopia 2013–2015. The maps illustrate the woreda-level prevalence of moderate-to-heavy intensity (MHI) infections for *Ascaris lumbricoides* (**a**), *Trichuris trichiura* (**b**) and hookworm (**c**) in 153,238 school-aged children, Ethiopia 2013–2015. The woreda prevalence is based on the number of MHI infections over the total number of children screened in a woreda. The classification of MHI infections are based on the World Health Organization criteria [4]. The source of the administrative boundaries is www.gadm.org/

The prevalence of MHI intensity of any STH infection was 2%. For the STH species separately, the proportions were 1.7%, 0.2% and 0.1% for *Ascaris*, *Trichuris* and hookworms, respectively. SNNPR showed the highest prevalence of MHI infections for each of the three STH helminths (*Ascaris*: 5.2%; *Trichuris*: 0.3% and hookworm: 0.2%). In seven out of 11 Regions/City Administrations no MHI infections were observed. Figure 2 illustrates the distribution of MHI infections for *A. lumbricoides* (Fig. 2a), *T. trichiura* (Fig. 2b), and hookworms (Fig. 2c) over the different woredas. The southern (SNNPR), south-western (Gambela, Oromiya and Benishangul-Gumuz) and northern (Amhara and Tigray) parts of the country have more infections of MHI. The variation in MHI infections was absent across both sexes and small across the different age categories. The results of the generalized linear mixed models confirmed the significant differences across Regions/City Administrations in the prevalence of STH infections of any intensity for each of the STH species. No significant differences in prevalence were observed for either sex or age. For the prevalence of MHI infections, the model did not convert.

### Prevalence of SCH infections of any intensity and MHI intensity

**Fig. 3:**
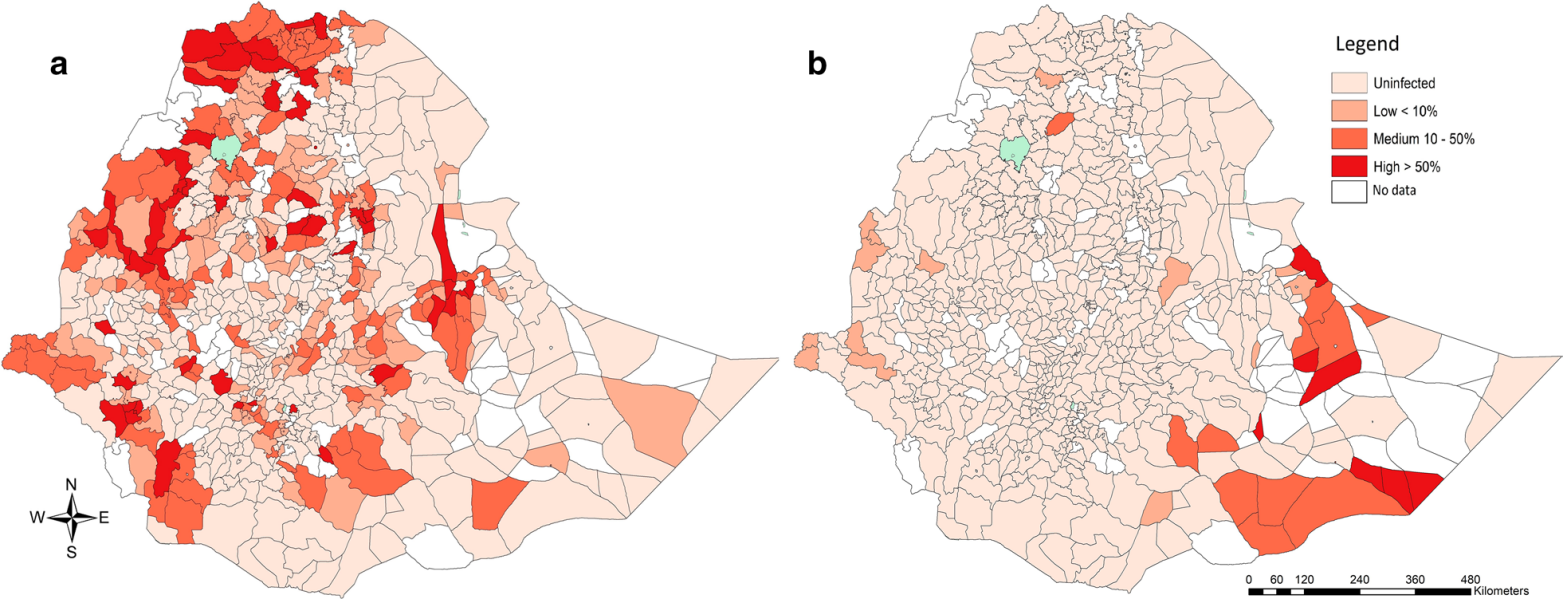
Woreda prevalence of *Schistosoma mansoni* and *S. haematobium* in school-aged children, Ethiopia 2013–2015. The maps illustrate the woreda prevalence of *S. mansoni* (**a**) and *S. haematobium* infections (**b**) in 153,238 (*S. mansoni*) and 112,217 (*S.* *haematobium*) school-aged children, Ethiopia 2013–2015. The woreda prevalence is based on the number of infections over the total number of children screened in a woreda. The source of the administrative boundaries is www.gadm.org/

The unweighted prevalence of SCH infections of any intensity and MHI intensity across the Regions/City Administrations, sex and age are summarized in Table 2. Across the subjects for whom both stool and urine were screened, the prevalence of infections of any SCH equalled 4.1%. Based on the SCH-specific data sets, *S. mansoni* was the most prevalent of both SCH species (3.5 *vs* 0.3%). As for STH infections, there was a large variation in prevalence across the different Regional States and City Administrations. For *S. mansoni*, the prevalence of any infection ranged from 0.1% (Addis Ababa City Administration) to 14.9% (Benishangul-Gumuz). *Schistosoma haematobium* was absent in 6 of the 11 Regions/City Administrations (Harari, Oromiya, SNNPR, Tigray, Addis Ababa and Dire Dawa) and highly prevalent in Somali (18.5%). The distribution of *S. mansoni* and *S. haematobium* infections across the different woredas is illustrated in Fig. 3. Overall, the variation in SCH infections across both sex and age was small. The prevalence of infections of any intensity was equally distributed over sex for both SCH species (*S. mansoni*: 3.4 *vs* 3.6%; *S. haematobium*: 0.3 *vs* 0.3%). Across the age categories, there was some increase over age infections, which was more apparent for *S. haematobium*. For this *Schistosoma* species, the prevalence ranged from 3.0% in SAC less than 9 years to 7.7% for children of 15 years of age. For *S. haematobium*, the variation in prevalence ranged from 2.9% for SAC of 11 years-old to 5.2% for SAC of 15 years-old.

**Table 2:** Variation in prevalence of schistosome infections across Regions/City Administrations, sex and age

	*Schistosoma mansoni* (%)			*Schistosoma haematobium* (%)			Any SCH (%)		
	*n*	Any intensity	MHI	*n*	Any intensity	MHI	*n*	Any infection	MHI
Region/City Administration									
Addis Ababa	2718	0.1	0.1	1990	0.0	0.0	1990	0.1	0.1
Afar	2377	0.3	0.1	1740	0.6	0.0	1740	0.9	0.1
Amhara	25,246	3.0	1.1	18,488	0.2	0.1	18,488	3.8	1.5
Benishangul-Gumuz	3109	14.9*	5.2	2277	5.4	0.0	2277	16.9	4.0
Dire Dawa	1701	6.2	2.6	1245	0.0	0.0	1245	7.4	3.3
Gambela	2977	9.2**	2.1	2180	3.8	1.3	2180	12.9	3.5
Harari	1804	6.0	2.9	1321	0.0	0.0	1321	6.8	3.3
Oromia	62,520	2.1	0.9	45,785	0.0	0.0	45,785	2.0	0.9
SNNPR	37,544	2.6	1.2	27,495	0.0	0.0	27,495	3.0	1.4
Somali	966	5.9	1.3	707	18.5	11.9	707	21.8	13.2
Tigray	12,276	10.8*	3.8	8990	0.0	0.0	8990	11.0	3.9
Sex									
Sex 0 (male)	76,385	3.4	1.3	56,061	0.3	0.1	56,061	3.9	1.5
Sex 1 (female)	76,853	3.6	1.4	56,156	0.3	0.1	56,156	4.1	1.6
Age (in years)									
5–9	1201	4.1	1.4	932	0.8	0.0	932	3.0	0.9
10	23,962	3.1	1.2	17,782	0.2	0.0	17,782	3.4	1.3
11	38,332	2.9	1.2	28,201	0.3	0.1	28,201	3.3	1.4
12	45,447	3.8	1.5	32,977	0.3	0.1	32,977	4.3	1.7
13	27,693	3.7	1.4	20,203	0.4	0.1	20,203	4.4	1.6
14	15,342	4.0	1.5	11,171	0.6	0.3	11,171	4.7	1.8
15	1261	5.2	2.1	952	2.3	1.8	952	7.7	4.1
Total	153,238	3.5	1.4	112,218	0.3	0.1	112,218	4.0	1.6

*Notes*: MHI: moderate-to-heavy intensity; comparisons to the baseline value (Region/City Administration: Afar; sex: sex 0; age = 12 years) that reveal an odds ratio significantly different of one are indicated by at least one asterisk

*P < 0.05, **P < 0.01, ***P < 0.001

**Fig. 4:**
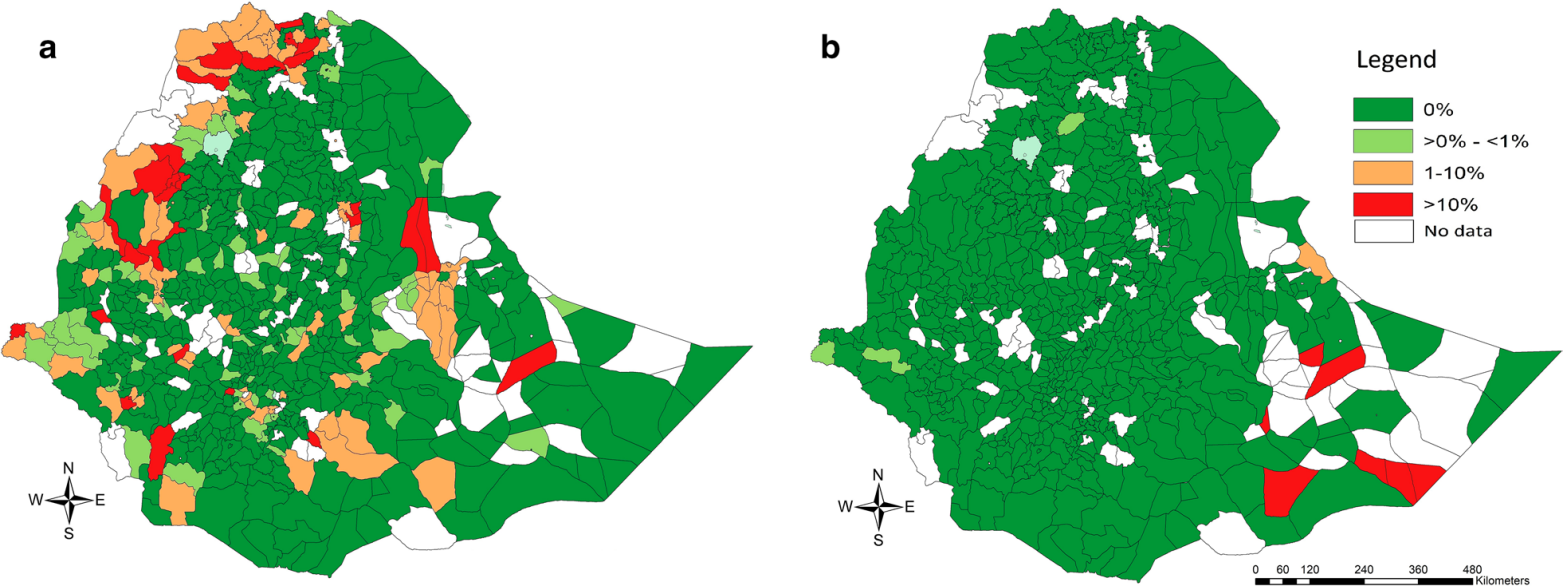
The woreda prevalence of moderate-to-heavy intensity schistsome infections in school-aged children, Ethiopia 2013–2015. The maps illustrate the woreada prevalence of moderate-to-heavy intensity *S. mansoni* (**a**) and *S. haematobium* infections (**b**) in 153,238 (*S. mansoni*) and 112,217 (*S. haematobium*) school-aged children, Ethiopia 2013–2015. The woreda prevalence is based on the number of MHI infections over the total number of children screened in a woreda. The classification of MHI infections are based on the World Health Organization criteria [4]. The source of the administrative boundaries is www.gadm.org/

Across the subjects for whom both stool and urine were screened, the prevalence of MHI infections of any SCH equalled 1.6%. Based on the SCH-specific data sets, the prevalence of MHI infections was 1.4% for *S. mansoni* and 0.1% for *S. haematobium*. The distribution of *S. mansoni* MHI infections across the Regions/City Administration followed a similar pattern as that observed for infections of any intensity, being high in Regions/City Administrations where infections of any intensity are prevalent (ranging from 0.1% in Addis Ababa City Administration and Afar to 5.2% in Benishangul-Gumuz). For *S. haematobium*, MHI infections were highly prevalent in 3 Regional States (Somali, Gambella and Amhara), of which Somali reported the highest prevalence (11.9%). The prevalence of *S. mansoni* and *S. haematobium* MHI infections across the different woredas is illustrated in Fig. 4. *Schistosoma mansoni* MHI infections were more prevalent in the west (Benishangul-Gumuz and Gambela) and the north of (Tigray), whereas MHI infections *S. haematobium* were more prevalent in the east of the country (Somali). The variation in MHI infections across sex was small for both SCH species. For age, the prevalence increased when children were older. The results of the generalized linear mixed models confirmed the significant differences across Regions/City Administrations in the prevalence of *S. mansoni* infections. No significant differences in prevalence were observed for either sex or age. For the prevalence of *S. haematobium* infections and MHI infections, the model did not convert.

### Mixed STH and SCH infections

Of the 112,218 SAC for whom both a stool and a urine sample was processed, 5776 children harboured infections with two or more helminth species. Mixed infections with two, three, four and five different helminth species were observed in 87.4%, 11.8%, 0.8% and 0.02% of these children, respectively. The different mixed STH and SCH helminth infections are summarized in Table 3. The most prevalent mixed infections were *Ascaris* infections mixed with either *Trichuris* or hookworms, accounting for more than 50% of the mixed infections.

**Table 3:** The mixed soil-transmitted helminth and schistosome infections

Mixed STH and SCH infections	*n* (%)
Five helminth species	1 (< 0.1)
All STH and SCH species	1 (0.02)
Four helminth species	48 (0.8)
*A. lumbricoides + T. trichiura +* hookworms + *S. mansoni*	45 (0.8)
*A. lumbricoides +* hookworms +*S. mansoni + S. haematobium*	2 (< 0.1)
*A. lumbricoides + T. trichiura +* hookworms + *S. haematobium*	1 (< 0.1)
Three helminth species	681 (11.8)
*A. lumbricoides + T. trichiura +* hookworm	500 (8.7)
*A. lumbricoides + T. trichiura + S. mansoni*	87 (1.5)
*A. lumbricoides +* hookworms + *S. mansoni*	49 (0.8)
*T. trichiura +* hookworm *+ S. mansoni*	34 (0.6)
*A. lumbricoides +* hookworms + *S. haematobium*	4 (0.1)
*A. lumbricoides + S. mansoni + S. haematobium*	3 (< 0.1)
*A. lumbricoides + T. trichiura + S. haematobium*	2 (< 0.1)
Hookworm *+ S. mansoni + S. haematobium*	2 (< 0.1)
Two helminth species	5046 (87.4)
*A. lumbricoides + T. trichiura*	2232 (38.6)
*A. lumbricoides +* hookworm	1371 (23.7)
*T. trichiura* + hookworm	462 (8.0)
Hookworm *+ S. mansoni*	380 (6.6)
*T. trichiura* + *S. mansoni*	287 (5.0)
*A. lumbricoides + S. mansoni*	228 (3.9)
*A. lumbricoides + S. haematobium*	50 (0.9)
Hookworm *+ S. haematobium*	19 (0.3)
*S. mansoni + S. haematobium*	15 (0.3)
*T. trichiura + S. haematobium*	2 (< 0.1)

### Recommended MDA strategy for STH and SCH infections

As outlined in the methods section, the woreda-level classification is based on the upper confidence interval of the woreda level estimate of ‘any STH’ or ‘any SCH’. Table 4 summarizes the endemicity of STH and SCH infections across the different woredas and the corresponding MDA strategy based on WHO guidelines [4]. For STHs, 178 out of 625 (28.5%) woredas require MDA once a year and 73 (11.7%) require MDA twice a year. For SCH, MDA once a year is recommended for 8 (1.3%) woredas, once every two years for 67 (10.7%) and once every three years for 190 woredas (30.4%). Large-scale treatment for STH and SCH was not warranted for 37 and 360 woredas, respectively.

**Table 4:** The classification of woredas into low, moderate and high endemic for soil-transmitted helminths and schistosome infections

Level of endemicity	Soil-transmitted helminths (%)	Schistosomes (%)
Absence of infections	37 (5.9)	360 (57.6)
Low	337 (53.9)	190 (30.4)
Moderate	178 (28.5)	67 (10.7)
High	73 (11.7)	8 (1.3)
Total	625 (100%)	625 (100%)

### Quality control of Kato-Katz thick smears

In total, 6042 Kato-Katz thick smear slides were re-examined at random by the team leaders. Overall, there was an agreement in test result in 91% of the positive cases (9% false negatives) and 98% of the negative cases (< 2% false positive cases). The numbers of false negatives were relatively high for *Trichuris* (8.7%) and low for hookworms (3.0%). Among the negative cases, there was an agreement in 93% of the cases. The agreement was high for *S. mansoni* (98.4%) and relatively low for *Ascaris* (93.7%).

## Discussion

The WHO has set the ambitious goal to cover at least 75% of SAC in need of treatment against STHs and SCH infections in all endemic countries by 2020 [4, 23–25]. In order to roll out an MDA programme, detailed disease maps are pivotal to ensure that areas most in need of MDA are covered, and to avoid large-scale deworming being initiated in areas were disease is rare or absent. Although studies highlight that Ethiopia is endemic for both STHs and SCH [5–7], up-to-date maps are currently missing. This study reports the results of the nationwide survey that was designed to provide accurate estimates of the disease distributions at the woreda level, which in turn will inform the national control program against STHs and SCH.

The results of the present study confirm that both STHs and SCH are prevalent in Ethiopian SAC. The overall prevalence for any STH in this population was estimated to be 21.7%, and 4.1% for any SCH in areas where both SCH species were assessed. To our knowledge, this will be the first large-scale survey conducted at national level. There have been many small-scale studies in different parts of the country that reported prevalence for STH and SCH [9, 11, 12, 14, 15, 26]. However, none of those studies were conducted at a national scale.

Our regional level prevalence for Amhara region is lower than the prevalence report by Nute et al. [8] across the different zones and woredas of the Amhara regional states (STH: 36.5; SCH: 6.9%) [8]. This difference might be explained by differences in diagnostic methods used (Kato-Katz thick smear *vs* formol-ether concentration), study population (SAC *vs* the entire community). Our 18.5% prevalence for *S. haematobium* in Somali regional state was in agreement with a previous report by Negussu et al. [13], but it remains unclear why this region is hyperendemic for SCH. However, our sampling strategy prioritized schools close to water bodies, and this region can have highly focal populations which are heavily concentrated around the relatively few rivers. This can provide pockets of high infection in areas that are conducive to snail vector breeding of bulinine snails. The relatively widespread distribution of *S. mansoni* and correlating limited distribution of *S. haematobium* infections in the country is striking. While this paper does not look to identify the underlying reasons, other researchers have identified the impact of altitude and climate on the distribution of the intermediate snail host species (*Biomphalaria* for *S. mansoni* and *Bulinus* for *S. haematobium*). For example, Kloos et al. [6] identified that most *S. mansoni* infections are found between 1300 m and 2000 m with areas outside that range unsuitable for *Biomphalaria pfeifferi* due to water temperature. Correspondingly, *S. haematobium* endemic areas are confined to low-lying areas below 800 m altitude, typically areas only found in Somali and Afar regions. These are in line with the results of the present study.

Our disease maps highlight that there are large geographical variations in prevalence of infections of any and moderate-to-heavy intensity (Figs. 1–4). This variation can be explained by a variety of factors, including but not limited to differences in climate, population density, and WASH facilities and usage. Alongside this study, associations between WASH and STHs/SCH infections were assessed and reported by Grimes et al. [21]. They concluded that better sanitation was associated with significantly lower *A. lumbricoides* infection intensity and borderline significant lower hookworm infection intensity. Better hygiene was associated with significantly lower hookworm intensities. However, no significant differences were observed when comparing sanitation and *S*. *mansoni* or *T. trichiura* infections and comparing hygiene and *A*. *lumbricoides* or *T*. *trichiura* infections. Figure 1 highlights that STH-endemic woredas are primarily located in the south, south-west, and north of the country. Dense population and agricultural activities in these areas potentially result in increased exposure to contaminated soil. In the eastern part of the country, infections are less abundant and this may be explained by a dry and arid climate, which does not favour the transmission of these parasites.

The success of the STH and SCH control programs can be measured by the reduction in moderate-to-heavy intensity infections. Ultimately, the WHO aims to eliminate these diseases as a public health problem, which is defined as a prevalence of moderate-to-heavy intensity infections less than 1%. The prevalence of overall moderate-to-heavy infections above 1% for both STH and SCH confirms the importance of the diseases as public health problems in Ethiopia [27]. Yet, it was striking that, despite the lack of large-scale MDA programmes and the abundance of STH and SCH infections, the prevalence of moderate-to-heavy intensity infections was already low (STH: 2%; SCH: 1.6%).

The study highlights that 59.3% and 26.7% of the woredas are endemic for STH (prevalence ≥ 20%) and SCH (prevalence ≥ 10%) at a level requiring MDA, meaning that 18 and 14 million SAC are in need of regular deworming through MDA campaigns for STH and SCH, respectively. The presence of STH and SCH co-infections shows the efficiencies that might be gained with an integrated control strategy. Indeed, in the past, integrated control has led to impressive reductions in prevalence and related morbidity [28]. In addition to integrating control against various parasites, the delivery of different types of parasite control might also be integrated. There are now recommendations to combine MDA with other interventions such as WASH to break parasite transmission and reach elimination [29].

The large areas of overlap in the distributions of the different STH species in Ethiopia could pose a challenge to future control programmes. Currently, the country is using mebendazole in all areas. However, previous studies indicated a differential efficacy between albendazole and mebendazole across STHs. Both drugs are equally efficacious against *Ascaris*, but albendazole is more efficacious against hookworms whereas mebendazole is more efficacious against *Trichuris* infections [30].

Following this national survey, Ethiopia has now distributed drugs for 4 years (2015–2019). Overall, the coverage has been high (> 75%) for both sets of diseases in all woredas [31]. In addition, 175 sentinel schools that represent the different levels of disease endemicity are currently periodically evaluated for the prevalence and intensity of both STH and SCH infections. These sentinel schools are also subjected to a variety of operational research activities in collaboration with national and international partners, with the aim of further improving the control program.

Ethiopia mobilized sufficient financial resources through a range of partners to support its nationwide survey to map the baseline distribution of both diseases. However, the cost of the survey was substantial, and there is a clear need to identify ways to reduce the cost of NTDs mapping, and to reduce the dependency on external partners. In response to this, we verified whether the examination of pooled samples rather than individual samples could be a potential cost-saving strategy in large-scale epidemiological surveys along this nationwide survey [32]. The outcome of this study highlighted that a pooled examination strategy reduced the laboratory time by 70%, but only resulted in an 11% cost reduction. Moreover, we also recommended pooling for the rapid assessment of infection intensity. However, further investigation is required to determine field procedures and statistical methods for optimal implementation of sample pooling for prevalence assessment.

This study has some limitations. First, the use of single instead of duplicate Kato-Katz thick smear has a clear impact on the prevalence results, as single Kato-Katz thick smear is known to be less sensitive than double Kato-Katz thick smear [33]. We opted for a single Kato-Katz for both practical and financial reasons. In general, we chose more children and fewer slides as better than fewer children and more slides. The impact of a single slide on estimates of infection intensity (mean FEC and proportion of moderate-to-heavy infections) is probably less pronounced, since mainly the lowest levels of egg excretion are missed [34]. Secondly, the school selection was biased towards SCH. This strategy was opted, because in contrast to STHs, SCH has a more focal distribution. Although this will have an impact on our estimates of SCH prevalence, we do not expect that this has a major impact on those for the prevalence of STH. Another aspect that may have an impact on the prevalence and hence the decisions made, is the sample size (5 schools per woreda and 50 children). Although this is the sample size recommended by the WHO, it has been shown that this strategy might not be optimal, resulting in unnecessarily administrating drugs or withholding administration of drugs to children in need of treatment, particularly for focally distributed diseases such as SCH [35]. Thirdly, the issues of security, fewer schools overall, and fewer children per school affected the sample size in some remoter areas, particularly in the Somali Region. This may have impacted the accuracy of the estimates in this area. For future surveys we recommend consideration of innovative methods to sample hard-to-reach, mobile and nomadic populations.

The objective of the mapping was to provide a robust estimate of the distribution of SCH and STH in Ethiopia, and hence the number of people that require treatment, with praziquantel (for SCH) and albendazole/mebendazole (for STH). It confirms Ethiopia as having a significant burden of both SCH and STH. In line with levels of infection found in neighbouring countries, such as Uganda [36], Tanzania [37] and Kenya [38]. Reducing SCH and STH infection in populous, high burden countries such as Ethiopia will be crucial for the global community to reach its ambitious 2020 and 2030 goals, under the umbrella of the WHO [1].

## Conclusions

From the regional and global prospective, our study provides optimal baseline information for Ethiopia to implement national SCH and STH programs, to enable it to reach the WHO’s 2020 goal, reaching 75% of SAC and 100% geographical coverage. The nationwide mapping confirms that Ethiopia is endemic to STH and SCH infections. The finding from this mapping warrants MDA to SACs living in areas that qualify for treatment. Further mapping initiatives that would provide useful programmatic information, would be the inclusion of preschool age children, adolescent and adult populations to scale up the control program, as well as fine-tuned mapping of hot spots for SCH.

## Supplementary information

**Additional file 1: Figure S1.** Flow diagram of the survey organogram. Survey management and supervision was cascaded in such a way that the Ethiopian Public Health Institute team led and oversaw the whole survey. Regional States supervisors supported their respective teams. The overall mapping implementation was checked by external supervisors.

## Data Availability

The data are owned by the Ethiopian Public Health Institute (EPHI) and are available from EPHI for those researchers who wish to analyse it. Please contact MT (melketadesse@yahoo.com) in the first instance.

## Abbreviations

EPG: eggs per gram stool
EPHI: Ethiopian Public Health Institute
FEC: fecal egg counts
FMoE: Federal Ministry of Education
FMoH: Federal Ministry of Health
MDA: mass drug administration
MHI: moderate-to-heavy intensity
NTDs: neglected tropical diseases
SAC: school-aged children
SCH: schistosomes
STH: soil-transmitted helminth
WHO: World Health Organization
