# Supplementary material for: National mapping of soil-transmitted helminth and schistosome infections in Ethiopia
Source: Parasit Vectors. 2020 Sep 1;13:437. doi: 10.1186/s13071-020-04317-6 (PMC7466696; doi:10.1186/s13071-020-04317-6)
Supplement: Supplementary file 1 — Additional file 1: Figure S1. Flow diagram of the survey organogram. Survey management and supervision was cascaded in such a way that the Ethiopian Public Health Institute team led and oversaw the whole survey. Regional States supervisors supported their respective teams. The overall mapping implementation was checked by external supervisors. [file 13071_2020_4317_MOESM1_ESM.docx]

Independent supervision and Technical support

(Ugandan VCD & KEMRI)

Mapping coordination

(By EPHI)

Regional coordinators

(Respective regions)

Daily Contact person

(Survey team leader)

**Additional file 1: Figure S1.** Flow diagram of the survey organogram. Survey management and supervision was cascaded in such a way that the Ethiopian Public Health Institute team led and oversaw the whole survey. Regional States supervisors supported their respective teams. The overall mapping implementation was checked by external supervisors.
